# Current Hospital Topics

**Published:** 1907-01-12

**Authors:** 


### HOSPITAL ADMINISTRATION.

### CONSTRUCTION AND ECONOMICS.

### CURRENT HOSPITAL TOPICS.

**Paying Wards in Special Hospitals.**

In September of last year the Committee of St. Peter's Hospital for Stone, Henrietta Street, W.C., expended about £1,200 on an additional ward containing six beds for the accommodation of paying patients. The step was taken in consequence of the income of the hospital not being sufficient to support more free beds. Patients are admitted to the pay ward at a fee sufficient to cover the cost of its upkeep. That upkeep is reckoned at 10s. a day for each patient. The step was taken with the approval of the honorary surgeons, who give their services gratuitously to the pay patients, as they do in the case of patients admitted to the public wards. We direct attention to this new departure on the part of a special hospital, for it is well calculated to meet the needs of a most deserving class of patients, who prefer to pay what they can when they are compelled to make use of one of the hospitals.

**Complimentary Dinner to Mr. P. J. Michelli, C.M.G.**

A committee has been formed, of which Mr. Perceval A. Nairne is the chairman, to suitably commemorate the honour of the Companionship of the Order of St. Michael and St. George conferred upon Mr Michelli a congratulatory dinner, at which ladies as well as gentlemen will be present, at the Trocadero Restaurant on February 15. It is further intended to present to Mr. Michelli a small memento as a tangible expression of the esteem in which he is held by a wide circle of friends and colleagues. The chair will be taken by Mr. Perceval A. Nairne. Mr. Michelli is widely and deservedly popular, and we welcome this movement to suitably commemorate so important an event in Mr. Michelli's career. The encouragement of eminent public service, rendered in the cause of hospitals and charities, by those who have had the best opportunity of appreciating its value, is a matter of the first importance to the well-being and efficient administration of these institutions.

**St. George's Hospital.**

The quarterly Court of Governors on January 11 had under consideration the question of abolishing the open board in favour of an elected house committee. The agenda paper shows once more that some of the older governors are born obstructives. Their method seems to be to move the adjournment or postponement of every proposal submitted to a meeting when they happen to be present. We dealt with this attitude of mind in a leading article on July 7, 1906, and there is really nothing to add to what was then fully stated as to the injury which may be unintentionally done by tactics of this description. The truth is, that, when a man attains an age when he is incapable of further active work, he should retire from all business, including that of hospital management. We heartily congratulate Mr. C. L. Todd, the retiring secretary, on the motion to grant him a pension of £550 a year on the completion of a period of forty-five years' service at the hospital. All superintendents, secretaries, matrons, and other efficient officers who have devoted their lives to the interests of a particular charity, should be able to look forward to a pension, on their retirement. It is rather remarkable that the opportunity offered by the Royal National Pension Fund, which includes hospital officials of all grades, has not been more generally utilised to provide such pensions on a federation basis.

**The Power of the Penny.**

The power of the penny has never been shown in a more striking manner than through the efforts of Mrs. Alfred Lucas on behalf of the Lowestoft Hospital. The success which has attended the League of Mercy, which in eight years has contributed £79,000, to the King's Fund, raised mainly by small subscriptions of 1s. a year and upwards, has evidently been taken to heart in Lowestoft. Mrs. Lucas found that there were 7,000 houses in Lowestoft, of which it was presumed 1,000 contributed subscriptions to the hospital directly or indirectly. Mrs. Lucas set herself to organise secretaries for districts and collectors for streets, with the object of asking the occupants of each of the 6,000 houses to contribute one penny a month to the hospital. In the first seventeen months £602 was raised from the 6,000 houses, being at the rate of 1½d. per month, or 1s. 6d. per annum. If a similar system were to be pursued in every urban community, the revenue from annual subscriptions to the local hospital might be easily and materially increased. It may be well to add a word of caution. In some places house-to-house collections have been pursued by all sorts of people for all sorts of objects, a system which is very justly resented by the householders. Each community might, very properly, in public meeting sanction a house-to-house collection, on Mrs. Lucas's plan, for the hospital, and at the same time resolve to request the police to put a stop to every street collection, for any other purpose, except it had been sanctioned by a public meeting of the residents.

**Bristol and its Hospitals.**

It has often been said that special efforts to raise funds for all the hospitals in a large city tend to diminish the contributions given to individual charities. We have held, as the result of many years' experience, that the view just stated, though the popular view, is opposed to the facts. The truth is, that, every special effort, in a given community, whether on behalf of all the institutions, or on behalf of one particular institution, has a most beneficial effect upon the revenue of all by extending the area of givers, and so tending in the end, to increase their receipts, from voluntary sources. A somewhat remarkable instance of the truth of this contention has occurred at Bristol. There, Sir George White, the President of the Bristol Royal Infirmary, has succeeded in raising a fund of £50,000 for the extension and reconstruction of the buildings of that institution. This effort, which has extended throughout the year, is now completed, and it might have been thought that it would have tended to diminish the contributions to the Hospital Sunday Fund, as the canvass for the Royal Infirmary was specially organised and continued with unabated vigour throughout the community. So far from this being the case, the amount received by the Hospital Sunday Fund, in Bristol, for the year, is the largest annual collection made, since the fund was inaugurated. Our congratulations are due not only to Sir George White, to the Lord Mayor and Council of the Hospital Sunday Fund, but to the inhabitants of Bristol, who have once more proved that, when a good object is properly brought before the notice of English people, it is sure to command their sympathies, and to receive their generous support.

